# Pediatricians’ Discomfort With Pre-exposure Prophylaxis (PrEP) for HIV Prevention: The Need for Greater Education

**DOI:** 10.7759/cureus.78826

**Published:** 2025-02-10

**Authors:** Matthew Grant, Kathryn Chenault, Jaylyn Waddell, Vicki Tepper

**Affiliations:** 1 Pediatrics, University of Maryland School of Medicine, Baltimore, USA

**Keywords:** adolescents, education, hiv, pediatricians, pre-exposure prophylaxis (prep)

## Abstract

Objectives

Despite being at a heightened risk of HIV, the use of pre-exposure prophylaxis (PrEP) among adolescents remains low, which may stem from access to this biomedical intervention and the comfort of providers caring for this priority population. Prior studies evaluating the comfort and knowledge of providers related to PrEP have focused on adult providers or been conducted prior to FDA approval of PrEP for adolescents. This study focuses on pediatric providers’ knowledge and comfort regarding counseling and prescribing PrEP to adolescents.

Materials and methods

Two anonymous surveys were administered to self-identified clinical providers treating pediatric patients at least part-time and practicing in Maryland in 2016 (n=118) and 2020 (n=60). General awareness, prior training, and personal clinical practice regarding PrEP were compared between respondent groups, as well as respondent self-reported comfort related to specific clinical scenarios to determine if pediatric provider knowledge and comfort have changed over time.

Results

General awareness of PrEP rose between the two surveys, with respondents reporting traditional education sources (continuing education, training, or colleagues) and non-traditional sources (television, the Internet, or social media). There was no change in self-reported comfort related to counseling and prescribing PrEP between the two surveys, and overall clinical knowledge remained low, with many respondents unable to identify necessary laboratory tests needed to begin medication safely.

Conclusions

Despite an increase in general awareness, provider comfort with PrEP did not increase over time. There is a need for ongoing education for pediatric providers to increase comfort and knowledge of biomedical HIV prevention to make it more readily available.

## Introduction

Adolescence is a development phase characterized by rapid physical, psychological, and social changes marking the evolution from a dependent child into an independent adult. Noteworthy, this period is the observed increase in risky behaviors (i.e., condomless sex, substance use), which hold potential long-term consequences and public health concerns (e.g., STIs, unintended pregnancy). Studies have shown that brain maturation continues well into early adulthood, with differential development of the limbic reward systems relative to prefrontal control systems [1] being a major contributor to increased risk-taking behavior and a lack of consideration of the consequences of actions in adolescents. Low rates of STI/HIV testing [2], condom use, and healthcare access among adolescents may stem from a lack of concern for long-term consequences.

Adolescents and young adults remain disproportionately affected by HIV. In 2019, 21% of the 36,801 new HIV infections in the United States occurred in youth aged 13-24. Adolescents in this age group are also the least likely of any age group to be aware of their infection or have a suppressed viral load [3]. Despite their elevated risk of acquiring HIV, studies have shown that the awareness and uptake of pre-exposure prophylaxis (PrEP) among all adolescents remains low [4-6]. Pediatric providers are uniquely poised to educate and offer PrEP to patients as an early intervention, given their ongoing role in their patients’ lives. The American Academy of Pediatrics (AAP) recommends yearly well-child evaluations throughout adolescence with a specific recommendation to provide opportunities for confidential discussions with the provider starting at age eleven. This recommendation, as well as recommendations for sexually transmitted disease counseling and screening based on risk, can be found in the AAP Bright Futures Guidelines [7]. These testing recommendations are reinforced by the Centers for Disease Control and Prevention [8]. The US Preventative Services Task Force recommends routine HIV testing of persons 13-64 years [9]; however, studies of adolescents have found that only approximately 9-10% of youth have been tested for HIV by the end of high school [10]. Many do not recall talking about HIV or hearing about PrEP from their provider [11], regardless of disclosing HIV risk factors.

PrEP is a highly effective biomedical intervention in the prevention of HIV [12]. Studies have shown that PrEP reduces the risk of HIV by 99% when taken consistently [13]. In 2018, the Food and Drug Administration (FDA) approved tenofovir disoproxil fumarate (TDF)/emtricitabine (FTC) for oral HIV PrEP use in adolescents >35 kg, following successful use for several years in the adult population. Tenofovir Alafenamide (TAF)/emtricitabine (FTC) was approved in 2019, and in December 2021, the FDA approved an extended-release injectable solution, cabotegravir [8].

Barriers to engaging patients in meaningful discussions around PrEP are multifactorial. Providers may feel discomfort in discussing sexuality or sexual risk behavior or perceive patient-related discomfort in the same topics [14]. Additionally, provider knowledge and training may present limitations, as providers may be unaware of recommendations for routine testing [15], be concerned about confidentiality related to age [16,17], or feel unprepared to provide recommendations regarding HIV prevention [18,19]. Provider discomfort in speaking about sexual health in general may additionally contribute to the low uptake of PrEP observed among adolescents and young adults [20]. The Purview Paradox, in which primary care providers may question their role vs. an HIV specialist as responsible for prescribing PrEP, may also play an important role for many clinicians [18]. Overall, healthcare utilization lessens during adolescence [21]. Therefore, the need to capitalize on communication during contact with healthcare providers around sexual health and prevention is critical. More significantly, key populations are at heightened risk, including sexual and gender minority groups, youths who engage in transactional sex, youths with discordant partners, and individuals who inject intravenous drugs [3], as data suggests these youths may be less likely to access care due to concern about stigma and discrimination, perhaps out of fear of judgment from their provider or clinical staff.

Prior studies evaluating the comfort and knowledge of providers related to PrEP have largely aimed at those caring for the adult population or were conducted prior to FDA approval of PrEP for adolescents [22,23]. Additional studies evaluated the comfort of adolescent medicine specialists [24,25] or of providers who specialize in working with people living with HIV [23,26]. This study focuses on pediatric providers’ knowledge and comfort with respect to talking about and prescribing PrEP to adolescents as an underutilized resource for HIV prevention and PrEP access among this priority population. This study aimed to determine how provider awareness of PrEP changed over time and if greater awareness correlated with (1) an increase in comfort in the management of adolescents seeking PrEP and (2) an increase in knowledge in clinical decision-making related to PrEP.

## Materials and methods

This study used two independent cross-sectional web-based surveys in 2016 and 2020. Prospective participants were recruited through email listservs, with surveys conducted via SurveyMonkey (Momentive Inc., San Mateo, CA, USA). Participants meeting inclusion criteria received information about the study and their rights as participants and consented to participate. Informed consent was implied based on participation in the electronic survey. To meet study eligibility, participants need to self-identify as a clinical provider treating pediatric patients at least part-time and practicing in Maryland. There were no exclusion criteria. The Institutional Review Board of the University of Maryland approved this study (approval number: HP-00091817, approval date: July 29, 2020).

Both surveys were structured in a mixed format, including binomial response (yes/no), multiple choice, and Likert scale (Table 1). One question was added to the 2020 survey prior to distribution, assessing sources of information related to PrEP. All the other questions were the same between the two surveys. Participants completed questions about demographics and personal experience with PrEP, self-assessed comfort levels with clinical scenarios involving caring for patients seeking PrEP-related services, and a short knowledge assessment related to laboratory monitoring and health assessment of those seeking PrEP. The average time for survey completion was less than 10 minutes.

**Table 1:** Knowledge (multiple choice) and self-reported comfort (Likert scale) questions used in 2016 and 2020 surveys PrEP: pre-exposure prophylaxis

Survey question
What patient history would help guide your decision to initiate a conversation about starting PrEP?
What counseling would you provide to your patient prior to starting PrEP?
What tests would you order prior to starting PrEP?
How frequently do you need to check labs on your patient taking PrEP?
How comfortable would you feel referring a patient to a specialty clinic for PrEP (i.e., knowing where to refer them?)
How comfortable would you feel prescribing PrEP to an adolescent patient today?
How comfortable would you feel monitoring labs for an adolescent patient on PrEP today?
How comfortable would you be looking up the treatment recommendations and then prescribing PrEP to patients in the future?
How comfortable would you be looking up the lab monitoring recommendations and then caring for patients on PrEP in the future?
Which providers should be responsible for being knowledgeable and offering PrEP to adolescents?

The level of training at the time of the survey (resident, fellow, or attending physician), the clinical focus of training (pediatrics, internal medicine-pediatrics, etc.), the current area of specialization (primary care, hospital-based medicine, specialty), and the year of graduation were collected at the time of the survey.

Participants were asked about general awareness of PrEP, prior experience in training with PrEP, personal clinical practice regarding PrEP, and sources of knowledge. Clinical comfort with PrEP was assessed via self-reported comfort-specific clinical scenarios involving PrEP using a five-point Likert scale. Respondents were asked to rate their comfort, with 5 being “very comfortable and confident” and 1 being “very uncomfortable and definitely wouldn’t do so.” The authors chose clinical scenarios based on relevance to various specialty practices. Respondents were additionally asked knowledge-based questions related to the routine delivery of medical care for patients seeking PrEP, including questions related to necessary laboratory monitoring and clinical counseling.

One hundred eighteen pediatric providers responded to the survey conducted in 2016. Sixty pediatric providers responded to the second survey in 2020. One response from the 2016 survey was excluded from the analysis as it was incomplete.

Statistical analysis

Statistical analysis was performed using SPSS Statistics version 26 (IBM Corp. Released 2019. IBM SPSS Statistics for Windows, Version 26.0. Armonk, NY: IBM Corp). Outcomes from the 2016 and 2020 surveys were compared to determine whether the survey time impacted participant response. Additional analysis of demographic responses was performed, including participation in training and year of graduation. Chi-square analyses were completed for all binomial questions/responses (i.e., Are you aware of PrEP?). Mann-Whitney U tests were conducted for the Likert scale questions assessing self-reported comfort on clinical scenarios to determine shifts in the distribution of answers. The Mann-Whitney U test is a non-parametric statistical test used to compare two independent groups when data does not follow a normal distribution, and it was ideal for comparing these two cross-sectional surveys.

## Results

A higher percentage of participants (56/60) in the 2020 survey reported being aware that providers were routinely prescribing antiretroviral medications as PrEP to prevent high-risk patients from contracting HIV than in the initial 2016 survey (58/117) (χ=33.13, p<0.0001). Participants in the 2020 survey noted the sources for learning about PrEP. The most common sources of education regarding PrEP included training (residency, fellowship) (26% responses), continuing education experience (grand rounds, departmental conferences) (21%), a medical journal (10.5%), or colleagues (12%). Responses also included non-traditional sources, including advertisements on television (12%), the Internet or social media (8.5%), or directly from patients (6%). Across all survey responses, current participation in a training program was associated with a higher likelihood of knowledge of PrEP, with 60% of trainees (68/114) versus 38% (24/63) of independently practicing providers reporting having heard of PrEP (χ=7.552, p=0.006).

Compared to the survey conducted in 2016, no statistical change was seen in the level of comfort among providers responding to clinical scenarios in the survey conducted in 2020. Mann-Whitney U tests were used to evaluate if the timing of survey participation impacted respondents' self-reported comfort in clinical scenarios. The results indicated that there was no significant difference between respondents in the 2016 and 2020 surveys. Results are shown in Table 2.

**Table 2:** Mann-Whitney U test results comparing the timing of survey participation versus self-reported comfort in clinical scenarios PrEP: pre-exposure prophylaxis

	2016 survey (N=93)	2020 survey (N=56)			
Respondent’s comfort	Mean rank	Mean rank	Z-value	Asymp. Sig (p)	r
Referring a patient to a specialty clinic for PrEP	77.77	69.13	-1.22	0.22	0.10
Prescribing PrEP to an adolescent patient today	78.75	68.77	-1.14	0.15	0.10
Monitoring labs for an adolescent patient on PrEP today	79.47	67.57	-1.69	0.09	0.14
Looking up the treatment recommendations and then prescribing PrEP to patients in the future	74.50	75.83	-0.19	0.85	0.02
Looking up laboratory monitoring recommendations and then caring for patients on PrEP in the future	75.74	73.78	-0.28	0.78	0.02

Across all survey responses, those in training were less comfortable referring patients to a specialty clinic for PrEP than those practicing independently. A Mann-Whitney U test was performed, indicating that those in training were significantly less comfortable than those out of training (U=1772, z=-3.78, p<0.0001, r=0.31). Mann-Whitney U tests were also used to evaluate if the level of training at the time of survey participation impacted self-reported comfort in other clinical scenarios. The results indicated that there was no statistically significant difference between those currently in training and those out of training in self-reported comfort related to scenarios involving prescribing PrEP, monitoring labs associated with PrEP, looking up treatment recommendations, and then prescribing PrEP in the future, or looking up lab monitoring recommendations and then caring for patients on PrEP in the future. These results are shown in Table 3.

**Table 3:** Mann-Whitney U test comparing participation in the training program at the time of survey participation versus self-reported comfort in clinical scenarios PrEP: pre-exposure prophylaxis

	Not currently in training (N=69)	In training (N=80)			
Respondent’s comfort	Mean rank	Mean rank	Z-value	Asymp. Sig (p)	r
Referring a patient to a specialty clinic for PrEP	88.32	62.43	-3.78	<0.0001	0.31
Prescribing PrEP to an adolescent patient today	73.99	75.88	-0.28	0.78	0.02
Monitoring labs for an adolescent patient on PrEP today	77.65	72.72	-0.72	0.47	0.06
Looking up the treatment recommendations and then prescribing PrEP to patients in the future	74.58	75.36	-0.12	0.91	0.01
Looking up laboratory monitoring recommendations and then caring for patients on PrEP in the future	77.46	72.88	-0.67	0.50	0.02

In knowledge-based questions, when asked to select patient history characteristics that might influence a decision to initiate a conversation about starting PrEP, participants noted patient sexual history, patient request, or a history of sexually transmitted disease as the top three options. When asked what laboratory testing would be appropriate prior to starting PrEP, a variety of answers were returned (Table 4): STI testing (60%), liver function test (50%), hepatitis panel (47%), basic metabolic panel (35%), and renal function panel (30%). Only 75% of respondents correctly indicated the need to order an HIV test prior to starting PrEP, with 32% of respondents indicating they did not know any appropriate orders.

**Table 4:** Laboratory tests recommended by participants as part of the initial evaluation of patients seeking PrEP on knowledge-based questions PrEP: pre-exposure prophylaxis, HIV: human immunodeficiency virus, STI: sexually transmitted infection

Laboratory test	Percent respondents
HIV	75%
STI testing (gonorrhea, chlamydia, syphilis)	60%
Liver function test	50%
Hepatitis panel	47%
Basic metabolic panel	35%
Renal function panel	30%
I don’t know	32%

Survey respondents were asked which providers should be responsible for being knowledgeable and offering PrEP to adolescents (Table 5). Results showed 100% of survey participants felt HIV/infectious disease specialists should be responsible, 93% adolescent medicine, 82% pediatric primary care providers (PCPs), 80% internal medicine PCPs, 75% family medicine PCPs, 53% OB/GYN providers, 25% ER physicians, and 25% hospitalists. No statistically significant differences were observed when compared to the same question asked in 2016.

**Table 5:** Providers/specialists are responsible for being knowledgeable and offering PrEP to adolescents, based on survey data PCPs: pediatric primary care, OB/GYN: obstetrician and gynecologist, ER: emergency room, PrEP: pre-exposure prophylaxis

Provider/specialty who should be responsible for PrEP	Percent respondents
HIV/infectious disease	100%
Adolescent medicine	93%
PCPs	82%
Internal medicine PCPs	80%
Family medicine PCPs	75%
OB/GYN providers	53%
ER physicians	25%
Hospitalists	25%

## Discussion

This study demonstrates the ongoing need for additional training for pediatric providers to provide PrEP. While general awareness of PrEP increased between survey time points, this study found no significant change in comfort in clinical scenarios related to prescribing or monitoring a patient on PrEP nor in knowledge necessary to safely prescribe and monitor the use of PrEP in a patient. More than 30% of the respondents could not correctly identify one laboratory test to recommend prior to the initiation of PrEP, and only 75% of respondents indicated that HIV testing would be among their initial laboratory tests, suggesting a significant knowledge gap.

Pediatric providers have not been included in prior large-scale PrEP education initiatives as most of the training and published information available to providers has been adult-focused [18,27]. While sexual health is incorporated into most training curricula, STI/HIV prevention mechanisms have not always been a focus of that education and may depend heavily on faculty training. More recently, there have been publications that provide guidelines for pediatric providers wishing to learn more and manage PrEP within their own practice. These articles provide concise management recommendations for the novice provider [28]; however, without broader training, PrEP will remain a preventative strategy viewed as the responsibility of HIV specialists, not general practitioners.

With adolescents remaining at disproportionate risk of HIV [3], findings from this study should serve as a call to action for healthcare providers in the HIV community. There is a substantial need for ongoing education for pediatric providers to increase comfort and knowledge with biomedical HIV prevention methods, including medications for PrEP. This should be integrated into the sexual health education provided as part of pediatric residency curricula and continuing education for established practitioners to ensure equitable access to patients and ensure every provider is comfortable offering this intervention. With nearly 30% of respondents noting some non-traditional forms of PrEP education (television, Internet, patient), there remain significant opportunities to integrate more structured education to improve provider knowledge into formal education.

These findings also highlight a significant gap in HIV prevention. Survey responses highlighted a common challenge in PrEP implementation: the diffusion of responsibility to specialty providers [29]. Though our study noted a high number of respondents also felt PCPs held responsibility, few felt ER physicians and/or hospitalists were responsible for being knowledgeable and offering PrEP, despite studies showing that adolescents may heavily rely on the ER, with those who do being at disproportionately higher risk for acquiring STIs, including HIV [30]. This perception did not significantly change between the two study time points, indicating a continued missed opportunity in provider education and awareness.

There are several limitations of this study. While every effort was made to recruit a broad cross-section of providers in both surveys, the study may not represent all pediatric providers. Participants were recruited in Maryland, limiting the generalization of the results to the wider pediatric community. It must also be acknowledged that the response rate was lower in the second survey despite recruitment from similar mechanisms. The COVID-19 pandemic may have impacted this, as the study was conducted in the spring of 2020, and the focus of many providers was drawn to the worldwide pandemic. Additionally, all information gathered was self-reported, including comfort levels. It may be very difficult to assess one’s comfort level in hypothetical situations accurately. For questions assessing knowledge, the study design did not ensure that respondents were not looking up information prior to or while completing the survey. Despite these limitations, this study effectively demonstrates the enduring need for additional resources and educational support for pediatric providers to effectively manage PrEP as a biomedical intervention in the prevention of HIV.

Future studies should focus on recruitment from a broad cross-sectional representation of pediatric providers, expanding recruitment across multiple states or regions of the United States. Additionally, including additional clinical scenario-based questions may strengthen the assessment of participant knowledge. Additional information on provider practices may support guideline development and implementation to improve access for adolescents seeking PrEP.

## Conclusions

Reducing HIV transmissions among the adolescent population will require employing all effective prevention strategies, including leveraging biomedical prevention interventions through primary care providers. Despite multiple years between surveys, this study found a continued diffusion of responsibility among pediatric providers. While general awareness of PrEP rose between survey time points, there remained significant discomfort with the administration of PrEP among respondents and a significant knowledge gap that impedes effective implementation in clinical practice. There is a critical need for additional education among pediatric providers to safely and effectively deploy PrEP in a manner accessible to adolescent patients. Focusing on STI/HIV prevention within pediatric and primary care training programs may help ensure pediatric trainees and clinical faculty develop the knowledge necessary to build access to this essential service.
